# Astrocyte Structural and Molecular Response to Elevated Intraocular Pressure Occurs Rapidly and Precedes Axonal Tubulin Rearrangement within the Optic Nerve Head in a Rat Model

**DOI:** 10.1371/journal.pone.0167364

**Published:** 2016-11-28

**Authors:** Shandiz Tehrani, Lauren Davis, William O. Cepurna, Tiffany E. Choe, Diana C. Lozano, Ashley Monfared, Lauren Cooper, Joshua Cheng, Elaine C. Johnson, John C. Morrison

**Affiliations:** Casey Eye Institute, Department of Ophthalmology, Oregon Health & Science University, Portland, Oregon, United States of America; Schepens Eye Research Institute, UNITED STATES

## Abstract

Glaucomatous axon injury occurs at the level of the optic nerve head (ONH) in response to uncontrolled intraocular pressure (IOP). The temporal response of ONH astrocytes (glial cells responsible for axonal support) to elevated IOP remains unknown. Here, we evaluate the response of actin-based astrocyte extensions and integrin-based signaling within the ONH to 8 hours of IOP elevation in a rat model. IOP elevation of 60 mm Hg was achieved under isoflurane anesthesia using anterior chamber cannulation connected to a saline reservoir. ONH astrocytic extension orientation was significantly and regionally rearranged immediately after IOP elevation (inferior ONH, 43.2° ± 13.3° with respect to the anterior-posterior axis versus 84.1° ± 1.3° in controls, p<0.05), and re-orientated back to baseline orientation 1 day post IOP normalization. ONH axonal microtubule filament label intensity was significantly reduced 1 and 3 days post IOP normalization, and returned to control levels on day 5. Phosphorylated focal adhesion kinase (FAK) levels steadily decreased after IOP normalization, while levels of phosphorylated paxillin (a downstream target of FAK involved in focal adhesion dynamics) were significantly elevated 5 days post IOP normalization. The levels of phosphorylated cortactin (a downstream target of Src kinase involved in actin polymerization) were significantly elevated 1 and 3 days post IOP normalization and returned to control levels by day 5. No significant axon degeneration was noted by morphologic assessment up to 5 days post IOP normalization. Actin-based astrocyte structure and signaling within the ONH are significantly altered within hours after IOP elevation and prior to axonal cytoskeletal rearrangement, producing some responses that recover rapidly and others that persist for days despite IOP normalization.

## Introduction

Glaucoma is a chronic optic neuropathy involving axon degeneration that begins at the level of the optic nerve head (ONH) [1, 2], and is the leading cause of permanent blindness worldwide [3]. Elevated intraocular pressure (IOP) is the only known modifiable risk factor for glaucoma [4, 5]. The question of what events link elevated IOP to eventual axon injury remains unanswered. Understanding early cellular and molecular responses to elevated IOP within the ONH will be critical to providing insights into possible neuroprotective strategies.

Astrocytes are glial cells that provide structural and physiologic support for ONH axons, and may serve as a link between elevated IOP and eventual axon degeneration [6]. The highly ordered and unique arrangement of ONH astrocyte extensions perpendicular to the axonal axis [7, 8], as well as their intimate contact with the extracellular matrix (ECM) [9], make them prime candidates for sensing and responding to mechanical strain from IOP fluctuations. Astrocytes are positioned along the connective tissues of the ONH, including the laminar beams in the primate ONH [10]. Astrocytes exhibit multiple extensions that enter and unsheathe axon bundles [7, 11, 12]. These astrocyte extensions further couple the meningeal vasculature to axons [13, 14], and are involved in neural development and synapse formation [15], metabolic and ionic support of ONH axons [16–18], facilitate mitochondrial transcellular degradation from retinal ganglion cell axons [19], and phagocytosis of myelin segments within the optic nerve [20, 21]. ONH astrocytes are also mechanosensitive [22–24] and dynamically respond to elevated IOP by retracting or reorienting their extensions [8, 12]. The reaction of ONH astrocytes to elevated IOP may lead to loss of structural and biochemical support of axons and eventual axon degeneration [25–28].

Structural and mechanical astrocytic response to elevated IOP likely involves integrin signaling and actin cytoskeletal dynamics [29, 30]. Integrins are transmembrane receptors that link the extracellular matrix (ECM) environment to the intracellular actin cytoskeleton and focal adhesion dynamics [31]. A large variety of integrin receptor subtypes have been identified within the human and primate ONH and are implicated in glaucomatous optic neuropathy [10]. Integrin receptor activity leads to direct activation of a number of intracellular kinases, including the focal adhesion kinase (FAK) and Src kinase family members [32]. Active FAK and Src kinase family members have been shown to be important regulators of cellular responses to injury in cultured astrocytes [33, 34].

The astrocytic actin cytoskeleton is important for astrocyte morphology [35], extension formation [35], migration [36, 37], communication and interaction with the ECM [9], and axonal neuroprotection [38]. In addition, nearly 100 ONH actin cytoskeletal and integrin-related gene expression patterns are altered with early glaucomatous injury in rodent models of glaucoma [39, 40]. Therefore, astrocyte integrin-based signaling and downstream actin cytoskeletal responses may provide a link between elevated IOP, astrocyte reactivity, and eventual axonal injury and degeneration. We hypothesize that actin-based astrocyte extension dynamics within the ONH are a sensitive indicator of astrocyte reactivity to elevated IOP, and that structural changes of astrocyte extensions are downstream of integrin signaling. If so, one would expect modulation in the activity of various downstream mediators of integrin receptors within the ONH upon IOP elevation, including FAK and Src kinases and their downstream targets, paxillin and cortactin, respectively. Here, we determine the astrocytic actin-based structural response, as well as major molecular responses downstream of integrin signaling, within the ONH after 8 hours of IOP elevation in a rat model.

## Materials and Methods

All animals were treated in accordance with the Association for Research in Vision and Ophthalmology statement for the use of animals in ophthalmic and vision research and all experimental methods were approved by the Oregon Health & Science University (OHSU) Institutional Animal Care and Use Committee. All surgeries were performed under isoflurane anesthesia, and all efforts were made to minimize suffering. Animals were housed and monitored according to OHSU’s Department of Comparative Medicine (DCM) guidelines, which included monitoring animals for illness, injury, or distress by a member of DCM on a daily basis.

### Animals and IOP Elevation

Thirty-one male Brown Norway rats (8–9 months old, 350-400g) were housed under alternating 12-hour cycles of light and dark. For IOP elevation, animals were maintained under general anesthesia using 2% isoflurane mixed with 100% oxygen, and kept at a temperature of 37°C using a water bath-controlled blanket. After topical 0.5% proparacaine hydrochloride ophthalmic solution application (Akorn, Lake Forest, Illinois, USA), the anterior chamber of one eye per animal was cannulated using a 31 gauge needle with care to avoid iris or lens trauma. The needle was linked to a reservoir filled with pH-balanced saline solution (BSS Plus, Alcon Laboratories, Fort Worth, Texas, USA) that was raised to produce a pressure of 60 mm Hg, as confirmed by a pressure sensor coupled with the infusion line (Harvard Apparatus, Holliston, Massachusetts, USA). Both eyes underwent external IOP measurements every 30 minutes during the period of IOP elevation (8 hours total) using a handheld tonometer (TonoLab; Icare Finland Oy, Espoo, Finland). Both eyes received topical proparacaine and topical BSS, alternating every 15 minutes to keep the ocular surface anesthetized and moist, respectively. Animals received a 1 mL subcutaneous injection of 0.9% saline every hour to maintain systemic hydration while under general anesthesia. After 8 hours of exposure to an IOP of 60 mm Hg, the saline reservoir was lowered to achieve an IOP of 20 for 5 minutes. Following this, the anterior chamber cannula was removed and animals were sacrificed either immediately (day 0), or allowed to survive for 1, 3, or 5 days after IOP normalization (7–8 animals in each of the following survival groups: day 0, 1, 3, and 5). During development of this model, IOP was monitored for up to a week after removal of the intraocular cannula in several additional animals, with no evidence of sustained IOP elevation at any point after the initial IOP elevation (data not shown). To assess axon injury, an additional group of animals underwent unilateral IOP elevation as above (4+ animals in each of the following survival groups: day 0, 1, 3, and 5). Controls included fellow un-injected eyes.

### Tissue Preparation, Actin Labeling, and Immuno-labeling, and Primary Antibody Validation

Prior to euthanasia, all animals were anesthetized under deep sedation with isoflurane and sacrificed by transcardial perfusion fixation with freshly prepared buffered 4% formaldehyde solution. In the group of animals used for axon injury assessment, the retrobulbar optic nerves were removed, post-fixed in 5% glutaraldehyde, embedded in plastic, sectioned and stained with toluidine blue (Electron Microscopy Sciences, Hatfield, Pennsylvania, USA), followed by light microscopy grading of morphologic axonal degeneration on a scale of 1 (no axonal injury) to 5 (axonal degeneration involving the entire nerve area). Section identities were masked and injury was graded by five observers and the grades were averaged as previously described [41, 42].

Perfusion-fixed eyes (including the ONH) were cryopreserved in 15% sucrose/phosphate buffered saline (PBS), followed by 30% sucrose/PBS, positioned for vertical longitudinal sectioning in Optimal Cutting Temperature support medium (Sakura Finetek, Torrance, California, USA), frozen in liquid isopentane cooled by liquid nitrogen, and kept on dry ice until ready for storage in a -80°C freezer. Frozen globes were cryo-sectioned at -20°C (5 μm thickness) onto gelatin-coated microscope slides (LabScientific, Inc., Highlands, New Jersey, USA). Sections closest to the sagittal (vertical midline) plane of the ONH were preferentially used for this study. The superior and inferior orientation of the ONH was determined based on the anatomic location of the central retinal vein and artery, located just inferior to the ONH [43].

Tissue sections were blocked with 1% bovine serum albumin (BSA) in PBS for 1 hour. Tissue sections were co-labeled with primary antibodies in 1% BSA/PBS at 4°C overnight, using antibodies against axonal tubulin (Tuj1; mouse monoclonal against βIII tubulin, 1:500 dilution, Covance, Seattle, Washington, USA), phosphorylated focal adhesion kinase (p-FAK; rabbit monoclonal against phosphorylated Tyr 397, 1:500 dilution, Life Technologies, Grand Island, New York, USA), active Src kinase (mouse monoclonal, raised against amino acid residues 519–536 of c-Src, 1:500 dilution, Life Technologies), phosphorylated paxillin (p-paxillin; rabbit polyclonal against phosphorylated Tyr 118, 1:100 dilution, Abcam, Cambridge, Massachusetts, USA), phosphorylated cortactin (p-cortactin; rabbit polyclonal against phosphorylated Tyr 421, 1:500 dilution, EMD Millipore, Billerica, Massachusetts, USA), or glial fibrillary acidic protein (GFAP, rabbit polyclonal; Dako, Carpentaria, CA, USA). Non-specific mouse or rabbit IgG antibodies (Sigma, Saint Louis, Missouri, USA) were used at similar concentrations to the respective antibodies above for negative control sections. Sections were washed with PBS at room temperature using three 5-minute cycles, followed by incubation with secondary fluorescent-labeled goat anti-mouse or goat anti-rabbit monoclonal antibodies (Alexa 488-labeled, Life Technologies) and actin filament marker tetramethylrhodamine (TRITC)-labeled phalloidin (Sigma) at a concentration of 1 μg/ml in 1% BSA/PBS at room temperature for 1 hour.[8, 44] After washing as above, cell nuclei were stained with 4',6-diamidino-2-phenylindole (DAPI) contained within the mounting media (Prolong Gold with DAPI, Life Technologies).

In order to validate the primary antibodies used for integrin-based immunofluorescence labeling, we used commercially synthesized blocking peptides (NeoBiolab, Woburn, Massachusetts, USA) to test for the specificity of the primary antibody. The blocking peptides used were the same sequence as the immunogenic peptide used for raising the primary antibody (per the commercial antibody supplier). The blocking peptide sequences were SSETDD[pY]AEII (p-FAK blocking peptide, corresponding to amino acids 390–401 of phosphorylated human FAK), HV[pY]SF (p-paxillin blocking peptide, corresponding to amino acids around the phosphorylation site of tyrosine 118 of human paxillin), LEDYFTSTEPQYQPGENL (active Src blocking peptide, corresponding to amino acid residues 519–536 of human c-Src), and PPSSPI[pY]EDAAPF (p-cortactin blocking peptide, corresponding to residues surrounding phosphorylated tyrosine 421 of mouse cortactin). The blocking peptides were dissolved in PBS at a stock concentration of 1 mg/ml. For primary antibody validation in our immunofluorescence labeling protocol, the primary antibodies (1:500 dilution) were incubated with the specific blocking peptide (1:500 dilution) in 1% BSA/PBS for 1 hour at room temperature. This mixture was then substituted as the primary antibody solution for labeling ONH tissue sections, with no other changes to the immunofluorescent labeling protocol described above.

### Microscopy and Image Analysis

Confocal images of the inferior and superior ONH (defined as a 75 x 75 μm area of the ONH just posterior to the termination of Bruch’s membrane, at either the ONH region adjacent to the central retinal vein [inferior] or opposite from the central retinal vein [superior]) were obtained using an FV1000 microscope (Olympus, Center Valley, Pennsylvania, USA), an UplanFLN 40x/NA1.30 oil objective, and 3.8x optical zoom. Images were captured using FV10-ASW version 4.0 software (Olympus) with laser wavelength settings of 405nm, 488nm, and 559nm at 1 μm/slice, using the same laser intensity and exposure time for all samples. FIJI image analysis software [45] was used to project a Z-stack of all acquired images per tissue section to visualize the maximum pixel intensity across the sample. The Directionality plugin feature of FIJI [46] was used to determine the mean orientation of actin bundles within the superior and inferior ONH, relative to anterior-posterior (A-P) axis (as defined by the longitudinal axis of axonal tubulin βIII labeling) [8]. To determine label intensity, mean fluorescence pixel intensity within images was calculated using FIJI software. In order to see if the brightest (i.e. most discernible) actin bundles within the ONH were altered after IOP elevation, we measured actin bundle lengths. For actin bundle length measurements, each Z-stack image was segmented into a grid with nine 25 x 25 μm sections, followed by identification of the 5 brightest actin bundles in each grid (a total of approximately 45 actin bundles per image). The identified actin bundles were measured using the ruler tool in the FV10-ASW software. In addition, for the purpose of normalization of actin filament measurements to a known cellular structure, additional length measurements of only actin filament in contact with nuclei were performed using the same grid methodology described above. Measured actin filaments that crossed between the designated grid margins were only counted once.

### Statistical Analysis

Statistical analysis was performed by two-way analysis of variance (ANOVA) for 5 independent groups and Dunnett’s multiple comparison testing was used to compare each experimental group to the control group (GraphPad Prism software, La Jolla, California, USA). Linear regression was performed using Prism software to assess for the presence of statistically significant deviation from a zero slope. All analyses were conducted using an alpha level setting of 0.05. Sample sizes (n) reflect the number of eyes used in each of the experimental (day 0, 1, 3, or 5) and control groups.

## Results

### Optic Nerve Injury After 8 hours of IOP Elevation is Minimal at Five Days Post Exposure

We first determined the level of morphologic optic nerve injury within the posterior, myelinated optic nerve from our model. IOP elevation during the 8 hour exposure was stable and comparable in all experimental groups (Table 1). The IOP of control eyes remained within a physiologic range during 8 hours of anesthesia. No significant morphologic injury was noted in control or experimental myelinated optic nerves, as assessed by light microscopy using a previously validated grading system [41, 42]. The longest surviving group after 8 hours of IOP elevation exhibited minimal morphologic optic nerve axon injury (day 5 injury grade of 1.1 ± 0.1 [n = 4] versus control injury grade of 1.0 ± 0.2 [n = 16]).

**Table 1:** Mean IOP Measurements During 8 Hours of IOP Elevation.

Group	n	IOP_Mean_ ± SD (mm Hg)	Injury Grade (n, p value relative to control)
**Control**	**30**	10.2 ± 1.6	1.0 ± 0.1 (16)
**Day 0**	**8**	58.7 ± 1.3	1.0 ± 0.0 (4, >0.05)
**Day 1**	**8**	58.7 ± 1.8	1.0 ± 0.0 (6, >0.05)
**Day 3**	**8**	57.2 ± 1.5	1.0 ± 0.0 (5, >0.05)
**Day 5**	**7**	57.1 ± 1.5	1.1 ± 0.1 (4, <0.01)

IOP = intraocular pressure (measured with a tonometer); n = number of eyes; SD = standard deviation

### ONH Astrocyte Extensions Change Orientation within Hours after IOP Elevation

Given the dynamic nature of astrocytes *in vivo* [8, 12] and in culture [35–37], we determined the actin-based structural response of the ONH astrocytes to 8 hours of IOP elevation in our rat model, using ONH actin bundles as a highly sensitive marker for astrocyte extensions [8]. We further differentiated the actin-based astrocyte responses of the superior and inferior ONH to elevated IOP, as the superior region of the ONH is more susceptible to axonal injury in models with chronic IOP elevation [6, 47–51], and is more susceptible to mechanical effects in models of acute IOP elevation [52].

**Fig 1:**
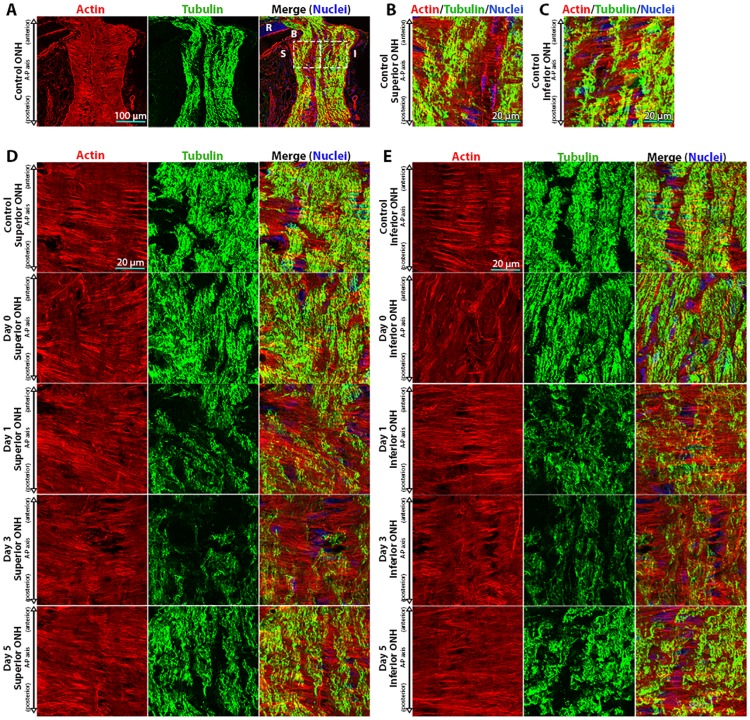
Actin-based astrocyte extensions change orientation within hours after intraocular pressure elevation. (A) Low magnification images of control optic nerve head (ONH) sections labeled for actin (TRITC-phalloidin), tubulin (Tuj1 anti-tubulin βIII antibody), and nuclei (DAPI). The left and right boxes in the merged image indicate the anterior superior and inferior regions of the ONH, respectively. (B, C) High magnification images of the superior and inferior regions of the ONH, as indicated by boxes in panel (A). Representative superior (D) and inferior (E) ONH regions from control eyes and eyes exposed to 8 hours of intraocular pressure (IOP) elevation, labeled for actin, tubulin, and nuclei. Day 0 eyes were immediately fixed after 8 hours of IOP elevation, while day 1–5 indicate the period of time the IOP was normalized post IOP elevation prior to fixation. A-P = anterior-posterior, B = Bruch’s membrane, I = inferior, R = retina, and S = superior.

**Fig 2:**
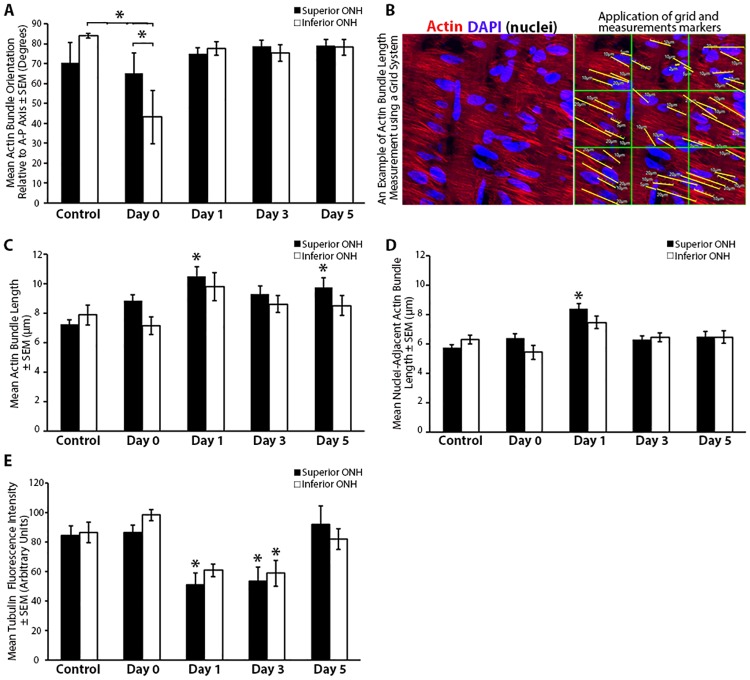
Quantitative analysis of actin-based astrocyte extension and axonal microtubule reorganization in response to elevated intraocular pressure. (A) Mean astrocytic actin bundle orientation relative to the anterior-posterior axis within the superior and inferior optic nerve head (ONH) of control eyes and eyes exposed to 8 hours of intraocular pressure (IOP) elevation. (B) An example of the superior ONH with actin and nuclear co-labeling, before and after application of a grid to allow for filament length measurement analysis. Note, in this example, only nuclear-adjacent actin bundles are identified and measured. (C) Mean length of actin bundles (with or without contact with a nucleus) within the superior and inferior ONH of control eyes and eyes exposed to 8 hours of IOP elevation. (D) Mean length of actin bundles in contact with a nucleus within the superior and inferior ONH of control eyes and eyes exposed to 8 hours of IOP elevation. (E) Mean axonal tubulin fluorescence intensity within the superior and inferior ONH of control eyes and eyes exposed to 8 hours of IOP elevation. Day 0 eyes were immediately fixed after 8 hours of IOP elevation, while day 1–5 indicate the period of time the IOP was normalized post IOP elevation prior to fixation. Error bars indicate standard error of the mean (SEM); * = p<0.05 by 2-way ANOVA and indicates statistically significant difference between control and experimental groups; n = 7, 7, 7, 8, and 6 for control, day 0, day 1, day 3, and day 5 groups, respectively. A-P = anterior-posterior.

Control superior and inferior ONH regions demonstrated highly ordered astrocytic actin bundles (Fig 1A–1C), which were nearly perpendicular to the A-P (axonal) axis of the optic nerve (70.3 ± 10.5° and 84.1° ± 1.3° in the superior and inferior ONH, respectively). There was minimal (if any) actin filament label along the A-P (axonal) axis, indicating limited contribution of actin filament signal from axons with the ONH, consistent with previous findings [8]. Immediately after exposure to 8 hours of IOP elevation (day 0), ONH astrocyte actin bundle orientation shifted to a more oblique and less-organized pattern relative to controls (Fig 1D and 1E). The reorientation of astrocyte actin bundles on day 0 was statistically significant for the inferior ONH relative to controls and to the superior ONH (Fig 2A). The structural organization of astrocyte extensions re-oriented back to baseline orientation within 1 day post IOP normalization in both the superior and inferior ONH, and remained at baseline orientation through day 5 post IOP normalization (Figs 1D, 1E and 2A).

Given the changes we observed in astrocyte bundle orientation with IOP elevation and the previously reported shortening of astrocyte processes in response to elevated IOP [12], we asked if astrocyte actin bundles underwent a change in length within the ONH in response to elevated IOP. Using a grid system to reduce selection bias (Fig 2B), we assessed actin bundle length throughout the superior and inferior ONH. In addition, in order to normalize actin bundle measurements to the cell body, we further assessed the length of only those actin bundles that were in contact with a nucleus. When assessing all actin bundles, control ONHs exhibited mean actin bundle lengths of 7.2 ± 0.3 μm and 7.9 ± 0.7 μm in the superior and inferior ONH, respectively (Fig 2C). In the experimental groups, ONH astrocytes exhibited an increase in mean actin bundle length within 1 day post IOP normalization relative to controls (10.5 ± 0.7 μm and 9.8 ± 0.9 μm in the superior and inferior ONH, respectively), which was also observed up to 5 days post IOP normalization (Fig 2C). This increase in mean actin bundle length in response to elevated IOP was statistically significant within the superior ONH on day 1 and day 5 post IOP normalization, relative to controls. When measuring only actin bundles that were in contact with a nucleus, control ONHs exhibited mean nuclear-adjacent actin bundle lengths of 5.7 ± 0.2 μm and 6.3 ± 0.3 μm in the superior and inferior ONH, respectively (Fig 2D). In the experimental groups, ONH astrocytes exhibited an increase in mean nuclear-adjacent actin bundle length within 1 day post IOP normalization relative to controls (8.4 ± 0.4 μm and 7.5 ± 0.4 μm in the superior and inferior ONH, respectively), which were statistically significant within the superior ONH relative to controls.

### Axonal Microtubule Filament Intensity within the ONH is Reversibly Altered after 8 hours of IOP Elevation

While no significant morphologic optic nerve axonal injury was noted in our model 5 days post IOP elevation, we asked if the axonal microtubule architecture within the ONH was altered after IOP elevation, given the intimate relationship between astrocyte extensions and axon bundles [8, 11]. Immediately after IOP elevation, axon-specific βIII tubulin label intensity (indicative of microtubule filaments) remained at levels comparable to controls (Fig 2E). However, 1 day post IOP normalization, the level of ONH axonal microtubule filament intensity was reduced, reaching statistically significant difference within the superior ONH region relative to controls (Fig 2E). This reduction in ONH axonal microtubule filament intensity was sustained 3 days post IOP normalization (reaching statistical significance in both the superior and inferior ONH) and returned to baseline levels within 5 days post IOP normalization (Fig 2E). Of note, this reversible reduction in ONH axonal microtubule filament intensity in response to IOP elevation was temporally preceded by astrocyte actin-based extension re-arrangement, which occurred immediately after IOP elevation (Fig 2A).

### IOP Elevation for 8 Hours Alters FAK Activity and Enhances Paxillin Phosphorylation within the ONH

**Fig 3:**
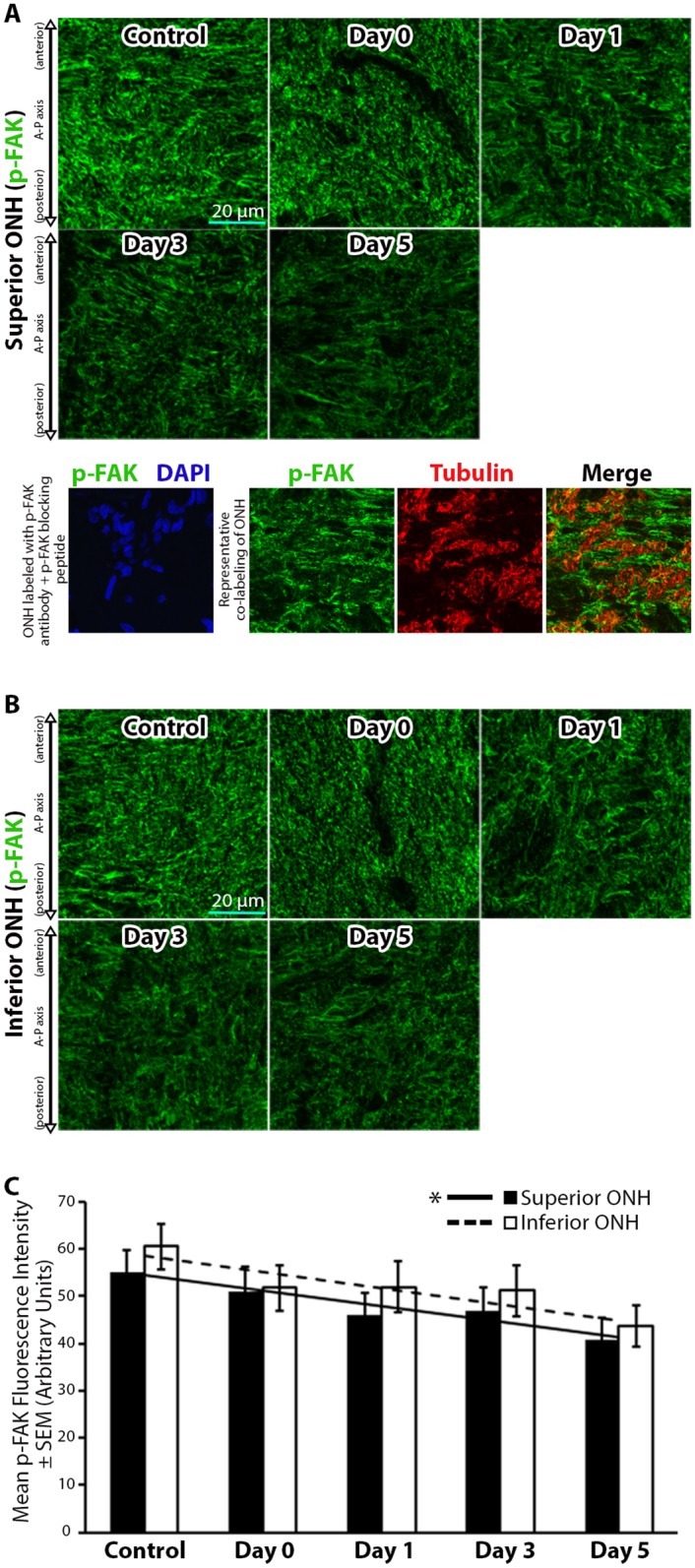
Focal adhesion kinase phosphorylation levels within the optic nerve head decrease steadily after intraocular pressure elevation. (A-B) Superior and inferior optic nerve head (ONH) sections labeled with anti-phosphorylated focal adhesion kinase (p-FAK) antibodies in control eyes and eyes exposed to 8 hours of intraocular pressure (IOP) elevation. The bottom panels in (A) include a validation of antibody specificity using a p-FAK specific blocking peptide (bottom left panel), as well as an example of co-labeling with p-FAK and axon specific anti-tubulin antibodies to demonstrate the non-axonal source of the majority of p-FAK label (bottom right panels). (C) Mean p-FAK fluorescence intensity within the superior and inferior ONH of control eyes and eyes exposed to 8 hours of IOP elevation. Day 0 eyes were immediately fixed after 8 hours of IOP elevation, while day 1–5 indicate the period of time the IOP was normalized post IOP elevation prior to fixation. Error bars indicate standard error of the mean (SEM). * = p<0.05 using linear regression analysis and indicates statistically significant difference in slope of the best fit trend line from a zero slope; n = 7, 6, 6, 7, and 6 for control, day 0, day 1, day 3, and day 5 groups, respectively. A-P = anterior-posterior.

Integrins facilitate mechanosensory cellular responses [53], and their activation [54–56] leads to phosphorylation of FAK (p-FAK; a kinase upstream of actin cytoskeletal remodeling) [57]. Given the significant and rapid actin-based astrocyte structural changes within the ONH in response to elevated IOP in our model, we asked if p-FAK levels were altered in response to elevated IOP. Baseline p-FAK levels were noted in control superior and inferior ONH sections (Fig 3A and 3B). Levels of p-FAK trended toward a reduction immediately post IOP elevation (day 0) and post IOP normalization on days 1–5 (Fig 3A–3C). While p-FAK levels for individual experimental groups post IOP elevation (day 0–5) were not statistically different from control levels, we did note a linear trend toward reduced levels of p-FAK post IOP normalization within the superior and inferior ONH (Fig 3C), which was statistically significant for the superior ONH (as defined by a best fit linear regression compared to a line with a zero slope, R^2^ = 0.77, p <0.05).

**Fig 4:**
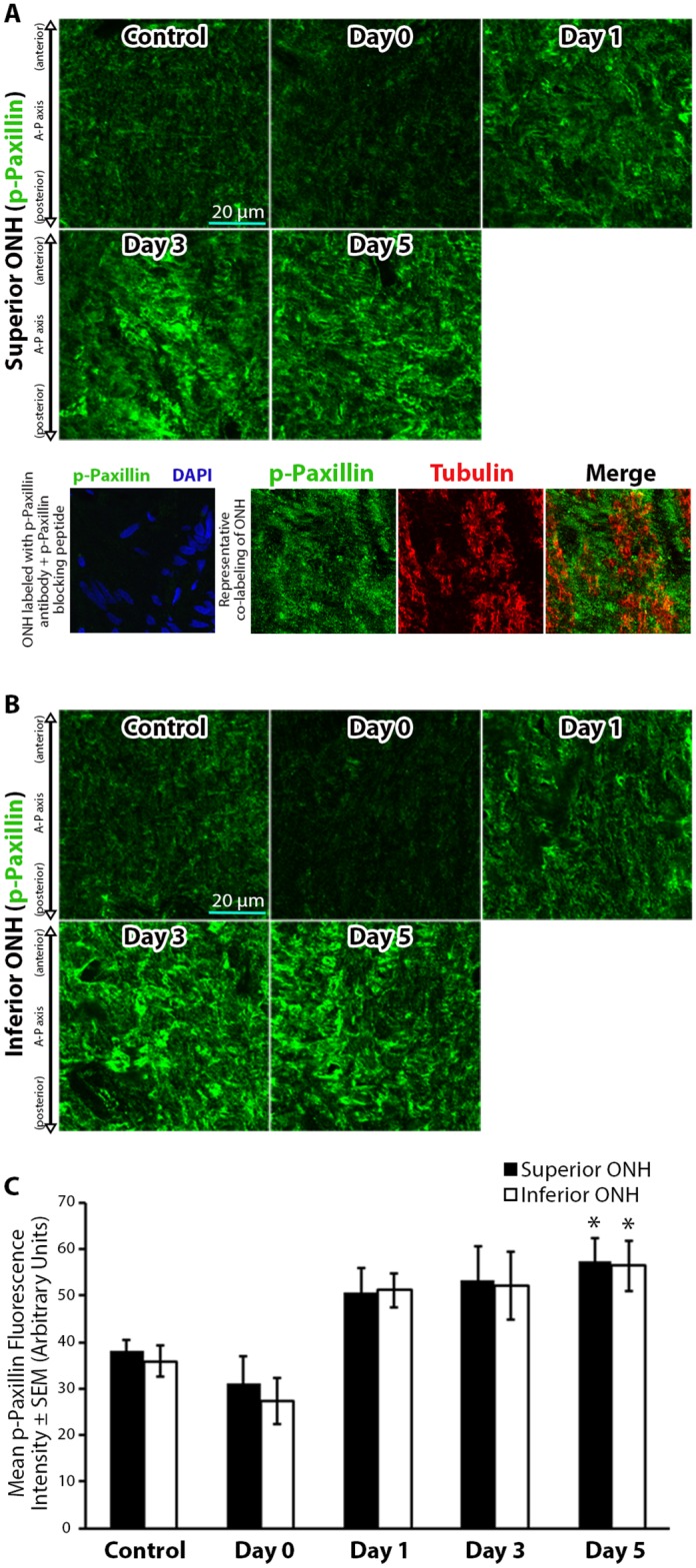
Paxillin phosphorylation levels within the optic nerve head increase and remain elevated after intraocular pressure elevation. (A-B) Superior and inferior optic nerve head (ONH) sections labeled with anti-phosphorylated paxillin (p-paxillin) antibodies in control eyes and eyes exposed to 8 hours of intraocular pressure (IOP) elevation. The bottom panels in (A) include a validation of antibody specificity using a p-paxillin specific blocking peptide (bottom left panel), as well as an example of co-labeling with p-paxillin and axon specific anti-tubulin antibodies to demonstrate the non-axonal source of the majority of p-paxillin label (bottom right panels). (C) Mean p-paxillin fluorescence intensity within the superior and inferior ONH of control eyes and eyes exposed to 8 hours of IOP elevation. Day 0 eyes were immediately fixed after 8 hours of IOP elevation, while day 1–5 indicate the period of time the IOP was normalized post IOP elevation prior to fixation. Error bars indicate standard error of the mean (SEM). * = p<0.05 by 2-way ANOVA and indicates statistically significant difference between control and experimental groups; n = 7, 7, 5, 7, and 7 for control, day 0, day 1, day 3, and day 5 groups, respectively. A-P = anterior-posterior.

Next, we asked if the level of phosphorylated paxillin (p-paxillin), a downstream target of p-FAK important for focal adhesion and actin cytoskeletal remodeling [58, 59], was altered in response to 8 hours of IOP elevation. Relatively low baseline levels of p-paxillin were noted in control ONHs (Fig 4A and 4B). Immediately after IOP normalization (day 0), p-paxillin levels appeared to be reduced (but did not a reach statistically significant change relative to controls). However, 1 day post IOP normalization, p-paxillin levels increased and reached significantly elevated levels 5 days post IOP normalization relative to controls (Fig 4A–4C).

### Src Kinase Target Cortactin is Phosphorylated within the ONH in Response to 8 Hours of IOP Elevation

**Fig 5:**
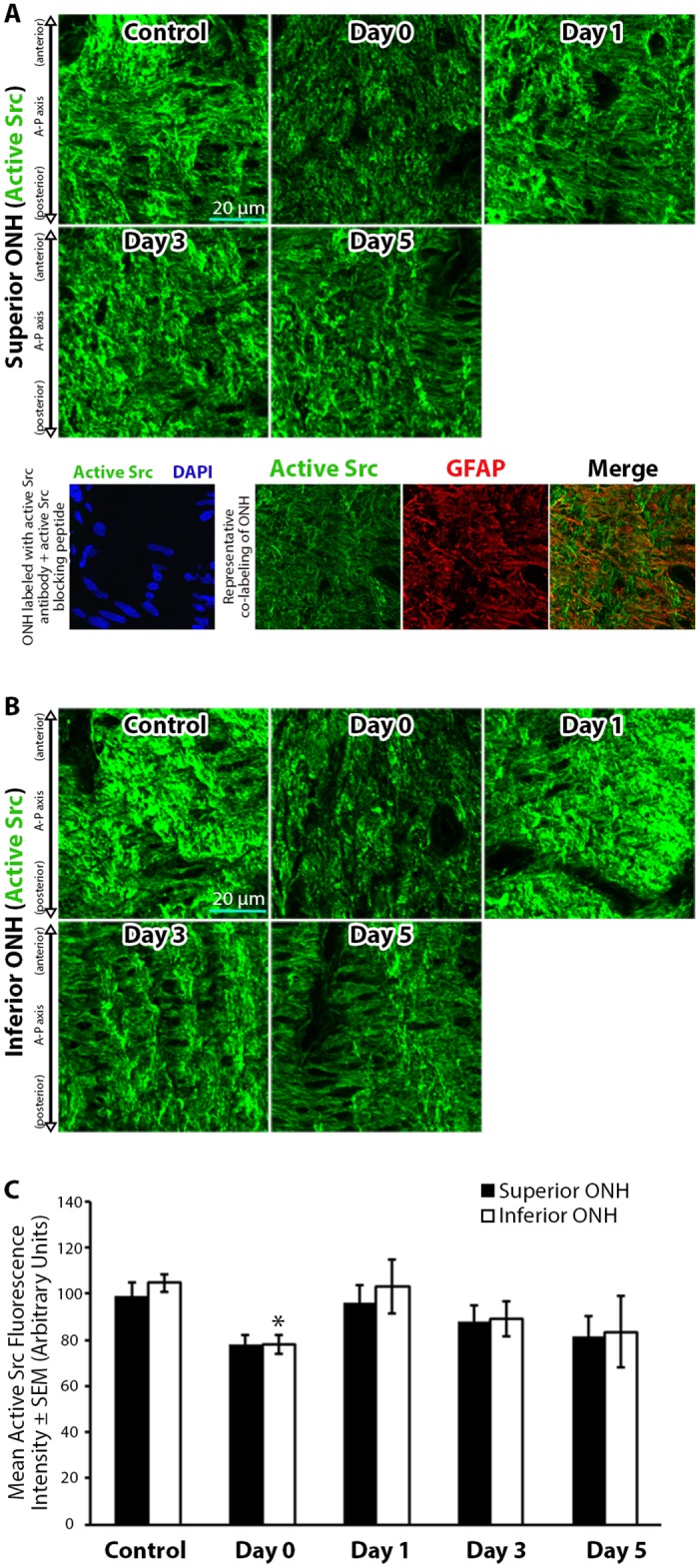
Active Src kinase levels within the optic nerve head are reduced immediately and transiently after intraocular pressure elevation. (A-B) Superior and inferior optic nerve head (ONH) sections labeled with antibodies against active Src kinase in control eyes and eyes exposed to 8 hours of intraocular pressure (IOP) elevation. The bottom panels in (A) include a validation of antibody specificity using an active Src specific blocking peptide (bottom left panel), as well as an example of co-localization of active Src with astrocyte specific anti-glial fibrillary acidic protein (GFAP) antibodies (bottom right panels). Note similar orientation and directionality of filaments labeled for active Src and GFAP. (C) Mean active Src fluorescence intensity within the superior and inferior ONH of control eyes and eyes exposed to 8 hours of IOP elevation. Day 0 eyes were immediately fixed after 8 hours of IOP elevation, while day 1–5 indicate the period of time the IOP was normalized post IOP elevation prior to fixation. Error bars indicate standard error of the mean (SEM). * = p<0.05 by 2-way ANOVA and indicates statistically significant difference between control and experimental groups; n = 7, 7, 6, 7, and 5 for control, day 0, day 1, day 3, and day 5 groups, respectively. A-P = anterior-posterior.

We next asked if Src kinase, an upstream regulator of actin polymerization [44, 60] and FAK phosphorylation [61], was active within the ONH. Active Src was abundantly found throughout control and experimental ONH sections (Fig 5A and 5B). Active Src levels were slightly reduced immediately after IOP elevation on day 0, reaching statistical significance within the inferior ONH relative to controls (Fig 5C). The level of active Src returned to control levels 1 day post IOP normalization, and remained unchanged 3 and 5 days post IOP normalization relative to controls (Fig 5C).

**Fig 6:**
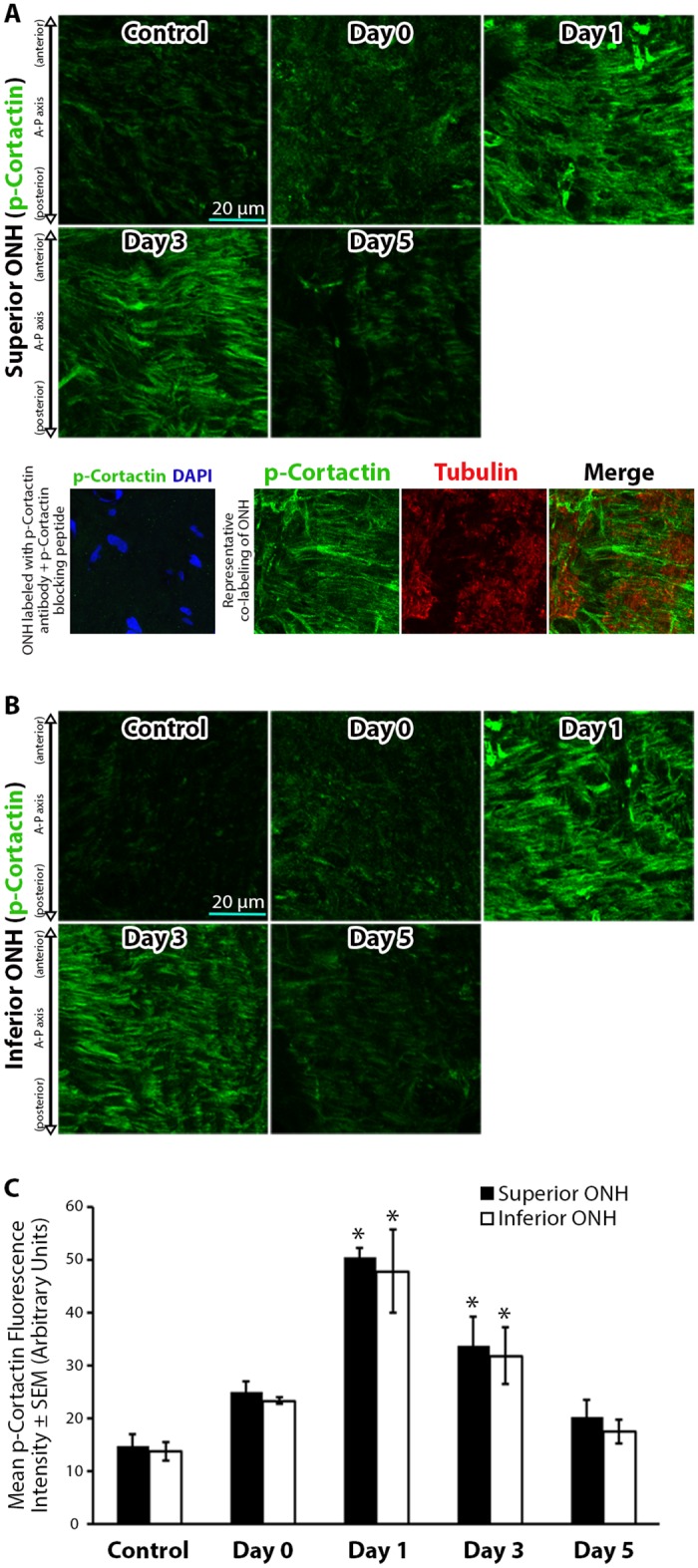
Cortactin phosphorylation levels within the optic nerve head increase in a reversible manner after intraocular pressure elevation. (A-B) Superior and inferior optic nerve head (ONH) sections labeled with anti-phosphorylated cortactin (p-cortactin) antibodies in control eyes and eyes exposed to 8 hours of intraocular pressure (IOP) elevation. The bottom panels in (A) include a validation of antibody specificity using a p-cortactin specific blocking peptide (bottom left panel), as well as an example of co-labeling with p-cortactin and axon specific anti-tubulin antibodies to demonstrate the non-axonal source of the majority of p-cortactin label (bottom right panels). (C) Mean p-cortactin fluorescence intensity within the superior and inferior ONH of control eyes and eyes exposed to 8 hours of IOP elevation. Day 0 eyes were immediately fixed after 8 hours of IOP elevation, while day 1–5 indicate the period of time the IOP was normalized post IOP elevation prior to fixation. Error bars indicate standard error of the mean (SEM). * = p<0.05 by 2-way ANOVA and indicates statistically significant difference between control and experimental groups; n = 7, 6, 7, 8, and 7 for control, day 0, day 1, day 3, and day 5 groups, respectively. A-P = anterior-posterior.

Given the abundant levels of active Src within the ONH, we asked if cortactin, a downstream target of Src kinase important for actin polymerization [44, 60, 62, 63], was phosphorylated in response to IOP elevation. Levels of phosphorylated cortactin (p-cortactin) were minimal in control ONHs, and remained statistically unchanged immediately after IOP elevation on day 0 (Fig 6A–6C). However, p-cortactin levels significantly increased and peaked 1 day post IOP normalization, and began to trend down 3 days post IOP elevation while remaining significantly elevated relative to controls (Fig 6A–6C). Levels of p-cortactin trended down further to control levels 5 days post IOP normalization (Fig 6A–6C).

## Discussion

Here, we present the first evidence of temporal and regional actin-based responses of ONH astrocytes to 8 hours of elevated IOP, which occur within hours of IOP elevation and prior to any significant morphologic evidence of axonal degeneration. In addition, while some of the structural and molecular responses of ONH astrocytes to elevated IOP are largely reversible within days after IOP normalization, we provide evidence of sustained changes in some molecular responses up to 5 days after IOP normalization. Our results also suggest a temporal separation of actin-based astrocyte structural re-orientation prior to tubulin-based axonal cytoskeletal reorganization.

Astrocytes are exquisitely sensitive to external mechanical stimuli [64], likely through sampling and interaction of integrin receptors with the ECM [29]. In culture, astrocytes exposed to external stretch respond by releasing adenosine 5’-triphosphate [22, 65] and endothelin [66]. IOP elevation *in vivo* induces rapid architectural changes of the ONH and ECM [67]. Given that astrocytes are in intimate contact with the ONH ECM, astrocytic extensions respond to IOP elevation by reorienting and retracting their extensions [8, 12, 68]. Here, we show the first evidence of rapid ONH astrocyte extension rearrangement immediately after 8 hours of IOP elevation, followed by re-orientation back to control levels within 1 day after IOP normalization. In addition, we noted an overall quantitative increase in the average length of actin-based astrocytic extensions 1 day after IOP normalization. The observed reversible changes in astrocyte extension orientation immediately after IOP elevation may imply acute shifts in the biomechanical forces present within the ONH tissue. Indeed, cells rich in actin (such as fibroblasts) are exquisitely sensitive to tissue strain and readily change orientation to counter stretch [69]. In addition, the observed reversible increase in astrocyte actin bundle length 1 day after IOP elevation, may imply that astrocyte response to local ONH mechanical tissue stress involves lengthening of extensions, which is consistent with observed reports of cultured astrocyte processes lengthening under mechanical stress [70].

Interestingly, ONH astrocyte architectural changes in response to short-term IOP elevation (30 mm Hg for 1 hour) in a murine model using glial fibrillary acid protein (GFAP) as an astrocyte marker [12], suggest astrocytic structural changes and recovery on the time scale of days to weeks after IOP elevation. Our results suggest a more rapid astrocyte rearrangement after IOP elevation, followed by a more rapid reorientation back to baseline levels after IOP normalization (both within hours). This difference may be explained by the level of IOP elevation and the time of exposure achieved in each model, and by the different astrocyte labeling techniques employed in the two models. Here, we use filamentous actin labeling, which is a highly sensitive, real-time indicator of astrocyte extensions [8]. In contrast, GFAP may not be present throughout all astrocyte extensions [71] and GFAP expression patterns have been shown to change with varying levels of tissue stress [72, 73].

The structural and molecular astrocytic changes we observed within the ONH occur prior to any significant morphologic axonal degeneration in the optic nerve. Indeed, other studies have demonstrated that a single short-term exposure to elevated IOP in rodent models does not cause long term effects on axon transport [74, 75]. However, our results indicate a reversible reduction in total axonal microtubule filament intensity within the ONH in response to 8 hours of IOP elevation, potentially due to a reversible depolymerization of microtubule filaments. This reduction in axonal microtubule filament intensity levels is delayed until 1 day post IOP normalization, sustained through 3 days post IOP normalization, and then recovers by 5 days post IOP normalization. This response of axonal microtubule filament rearrangement occurs later than the actin-based astrocyte extension re-arrangement that we observed immediately after IOP elevation. The temporal relationship between these two findings support the possibility that astrocyte architectural re-arrangement in response to IOP elevation may precede axonal injury [8, 76], and may possibly have a causal role in axon degeneration. It is conceivable that repeated or chronic astrocyte architectural changes in response to elevated IOP result in the loss of optimal astrocyte structure and function, which further lead to permanent and irreversible structural changes of axonal microtubule cytoskeleton. Axonal microtubule cytoskeletal disruption would impede axonal transport of cellular cargo and mitochondria [77, 78], and may accelerate eventual axonal degeneration. Indeed, astrocyte morphology alterations have been shown to correlate with axon loss in rodent models of glaucoma [68]. Thus, astrocyte response to elevated IOP within the ONH may be the earliest event in IOP-mediated cellular responses, which result in subsequent chronic changes to astrocyte structure and function that are incompatible with axonal support. This hypothesis remains to be tested in future studies.

Our results indicate a subtle, but significant differential structural rearrangement of actin-based astrocyte extensions within the superior and inferior region of the ONH. In our study, the structural rearrangement of astrocyte extensions in response to elevated IOP are most significant in the inferior ONH immediately after IOP elevation (Fig 2A, day 0). Differential susceptibility to axonal damage between the superior and inferior ONH regions in rodent glaucoma models has been described, and indicates a predilection for superior ONH axon injury [6, 47–51]. The preferential rearrangement of inferior ONH astrocyte extensions in our model may still be consistent with reports of superior ONH susceptibility to axonal injury. As reported by Sun et al [12], rodent astrocyte extensions span the entire transverse diameter of the ONH. As IOP-induced biomechanical strain within the ONH [79] may preferentially be transmitted to astrocytes in the superior ONH, one would expect these astrocytes to be more reactive. Rodent astrocytes cell bodies located within the superior ONH have relatively long, distal extensions spanning to the inferior ONH [12]. These longer, distal extensions are the first to retract and rearrange upon astrocyte reactivity after elevated IOP [12], likely leading to the inferior ONH structural changes observed here. Further investigations into this hypothesis will require detailed finite element modeling of the ONH in response to mechanical strain [80–82], including the contribution of highly ordered actin-based astrocyte extensions to these models.

**Fig 7:**
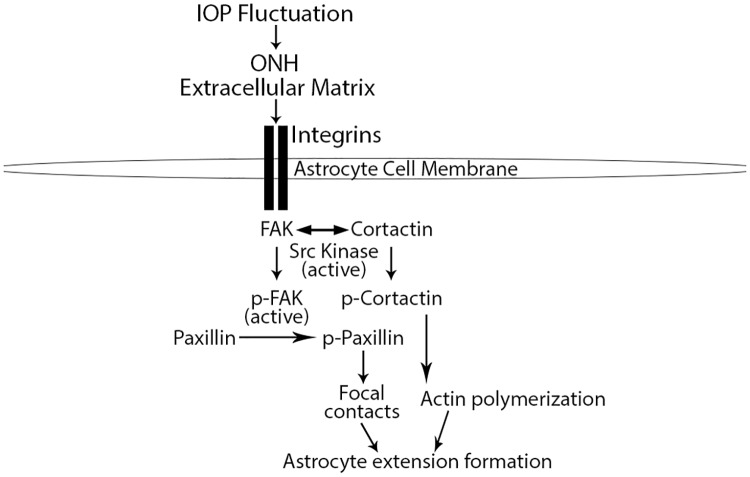
Model of optic nerve head astrocyte signaling downstream of integrin activation in response intraocular pressure fluctuation. Physiologic and experimental intraocular pressure (IOP) fluctuation within the optic nerve head (ONH) results in tissue strain and extracellular matrix (ECM) stretch. Interaction between the ECM and integrin receptors on astrocyte cell membranes, leads to integrin activity, which phosphorylate focal adhesion kinase (p-FAK). Active p-FAK can target paxillin for phosphorylation, which is important for focal contact/adhesion formation. FAK phosphorylation also allows for cortactin phosphorylation through active FAK and Src kinase. Phosphorylated cortactin enhances actin polymerization. Increased focal contact formation and actin polymerization are required for dynamic astrocyte extension formation and maintenance within the ONH.

We hypothesize that the rapid structural changes we observed in ONH astrocyte extension orientation in response to IOP elevation are downstream of integrin signaling (Fig 7). Integrins are cell membrane receptors that interact with the ECM and link extracellular signaling with the intracellular actin cytoskeleton [31]. In addition, integrins are distributed throughout the ONH along the margins of ECM beams and within the glial column [10]. Our results support a rapid intracellular cascade of signaling downstream of integrin receptors involving FAK and Src kinase, and more importantly their respective targets paxillin and cortactin, which have a direct role in actin cytoskeletal remodeling.

First, in control eyes under normal IOP fluctuations, active p-FAK and p-paxillin are present within the ONH. This likely indicates a dynamic and homeostatic interaction between the ECM and the intracellular actin cytoskeleton (through basal integrin activity) to optimize astrocytic extensions in their baseline structural steady state. In this scenario, cortactin remains inactive, potentially through sequestration by the unphosphorylated/inactive portion of the FAK population, which renders cortactin inaccessible for phosphorylation by both FAK and Src kinase [63, 83].

Immediately after 8 hours of IOP elevation (day 0), p-paxillin levels remain unchanged relative to controls. As p-paxillin is necessary for focal contact formation [58], this likely indicates no additional focal contacts formation by astrocyte extensions on day 0 relative to baseline. Un-elevated levels of p-paxillin immediately after IOP elevation may allow for astrocyte extension rearrangement, as observed in this study.

Within 1 day after normalization of IOP, p-paxillin and p-cortactin levels increase within the ONH relative to controls. Phosphorylation of paxillin and cortactin have been shown to enhance focal adhesion formation [32] and actin polymerization [60], respectively, while p-cortactin is necessary for optimal actin dynamics [44]. Taken together, the above changes after IOP normalization may allow for astrocyte extension reformation through enhanced actin polymerization and establishment of new focal contacts. Interestingly, the levels of two of the upstream kinases responsible for p-paxillin and p-cortactin phosphorylation (namely, FAK and Src kinase, respectively), do not significantly increase within the ONH in response to IOP elevation. Indeed, ONH p-FAK levels trend toward lower levels post IOP normalization in our model. This may highlight the importance of other mediators of paxillin and cortactin phosphorylation in response to elevated IOP within the ONH, including the role of other kinases [84–86], phosphatases [87, 88], adaptor proteins [89, 90], and the cellular localization of paxillin and cortactin [91, 92].

Several days after IOP normalization, some of the molecular markers of integrin signaling and actin dynamics have returned to baseline levels within the ONH in our model. However, p-paxillin levels within the ONH continue to remain significantly elevated 5 days post IOP normalization relative to control levels, while ONH p-FAK levels steadily decline post IOP normalization. These results are particularly significant as sustained molecular changes within astrocytes after IOP elevation may play a role in axonal injury in repeat or chronically-sustained IOP elevation models. In such a scenario, repeated IOP elevations may result in additional or synergistic astrocytic structural and molecular changes, which may render astrocytes more reactive to future IOP elevations and therefore less supportive of axons. Indeed, human eyes with greater glaucomatous injury appear to have increased susceptibility to IOP elevation [93], while some patients with glaucomatous injury continue to lose vision despite reduction of their intraocular pressure [94].

In conclusion, 8 hours of IOP elevation in a rat model leads to rapid structural and molecular changes within ONH astrocytes prior to morphologic axon degeneration. These changes are likely mediated through integrin receptors, which act as a link between the ECM and intracellular actin cytoskeleton. While some of the structural and molecular astrocyte changes in response to 8 hours of IOP elevation return to baseline after IOP normalization, evidence of sustained molecular changes within the ONH offers a possible clue into the pathogenesis of axon injury in chronic and repeated IOP elevation, as characterized in primary open angle glaucoma.
